# A Mitochondrial Plasma Proteomic Signature Identifies Metastatic Chromophobe Renal Cell Carcinoma

**DOI:** 10.3390/cancers18061032

**Published:** 2026-03-23

**Authors:** Clara Steiner, Tiegang Han, Steven Safi, Wafaa Bzeih, Hadi Mansour, Eddy Saad, Jessica F. Williams, Michelle S. Hirsch, Vinay K. Giri, Liliana Ascione, Yehonatan Elon, Adam P. Dicker, Yan Tang, Toni K. Choueiri, Elizabeth P. Henske, Wenxin Xu

**Affiliations:** 1 Dana-Farber Cancer Institute, Boston, MA 02215, USA; clara.steiner@medizin.uni-leipzig.de (C.S.); liliana_ascione@dfci.harvard.edu (L.A.);; 2 Department of Urology, University Hospital Leipzig, 04103 Leipzig, Germany; 3 Brigham and Women’s Hospital, Boston, MA 02115, USA; than5@bwh.harvard.edu (T.H.); hmansou1@hfhs.org (H.M.); ytang11@bwh.harvard.edu (Y.T.); 4 Henry Ford Hospital, Detroit, MI 48202, USA; 5 Beth Israel Deaconess Medical Center, Boston, MA 02215, USA; 6 Spencer Fox Eccles School of Medicine, University of Utah, Salt Lake City, UT 84132, USA; 7 Harvard Medical School, Boston, MA 02115, USA; 8 Division of Early Drug Development, European Institute of Oncology (IEO), Istituto di Ricovero e Cura a Carattere Scientifico (IRCCS), 20139 Milan, Italy; 9 Department of Oncology and Hematology (DIPO), University of Milan, 20122 Milan, Italy; 10 Oncohost, Binyamina-Giv’at Ada 3057324, Israel; 11 Department of Radiation Oncology, Sidney Kimmel College of Medicine, Sidney Kimmel Comprehensive Cancer Center, Thomas Jefferson University, Philadelphia, PA 19107, USA

**Keywords:** chromophobe renal cell carcinoma, mitochondria, metabolic reprogramming, LASSO, SomaScan, proteomics

## Abstract

Background: Chromophobe renal cell carcinoma (ChRCC) is characterized by the accumulation of abnormal mitochondria, a high rate of mitochondrial DNA (mtDNA) mutations, and altered oxidative metabolism. There are no existing circulating biomarkers to distinguish metastatic ChRCC from clear cell renal cell carcinoma (ccRCC). Methods: High-throughput plasma proteomic profiling using the SomaScan platform was performed in 18 ChRCC (including 16 metastatic ChRCC) and 197 metastatic ccRCC patients. Data were harmonized to generate a unified 7K-protein matrix. Results: Differential expression analysis was performed using limma (version 3.62.2). Of 7272 quantified human plasma proteins, 209 were differentially expressed between ChRCC and ccRCC. Upregulated proteins in ChRCC included essential β-oxidation enzymes such as ECH1 (enoyl-CoA hydratase 1) and ECI1 (enoyl-CoA delta-isomerase 1), suggesting increased long-chain fatty acid degradation. Creatine and energy-buffering pathways were also represented, with increased CKMT1A (Creatine Kinase, Mitochondrial 1A) in ChRCC. KIM-1 (Kidney Injury Molecule-1) and leptin were lower in ChRCC, consistent with the known upregulation of these proteins in ccRCC. Pathway enrichment analyses revealed an overrepresentation of mitochondrial protein degradation, fatty acid β-oxidation, and respiratory electron transport in ChRCC, suggesting that ChRCC sheds a unique mitochondrial signature into the peripheral circulation. A bootstrap-based LASSO logistic regression restricted to upregulated mitochondrial proteins in ChRCC vs. ccRCC consistently selected ECI1 and CKMT1A. The LASSO model achieved an AUROC of 0.964. Conclusions: Compared to ccRCC, the plasma proteome of metastatic ChRCC is dominated by mitochondrial metabolic enzymes, revealing a systemic metabolic phenotype strikingly aligned with the known histologic accumulation of abnormal mitochondria in ChRCC cells.

## Simple Summary

Chromophobe renal cell carcinoma is a rare subtype of kidney cancer that differs biologically from the more common clear cell kidney cancer. However, there are currently no blood-based tests that can reliably distinguish these tumor types, particularly among patients with advanced disease. In this study, we analyzed thousands of proteins circulating in the blood of patients with these two cancer types to identify patterns that could help differentiate them. We found that patients with chromophobe kidney cancer show higher levels of proteins involved in mitochondrial energy metabolism. This could reflect the well-known accumulation of abnormal mitochondria in these tumors. Using these proteins, we developed a simple two-marker model that distinguishes chromophobe from clear cell kidney cancer. These findings suggest that protein signatures in the blood might help improve the identification of chromophobe kidney cancer and give new insights into its metabolism and tumor biology.

## 1. Introduction

Chromophobe renal cell carcinoma (ChRCC), the third most common subtype of renal cell carcinoma (RCC), is histologically marked by pale to eosinophilic cytoplasm, perinuclear halos, and massive accumulation of mitochondria [1]. It can occur sporadically or in association with the hereditary tumor syndromes Birt–Hogg–Dubé syndrome (BHD) and tuberous sclerosis complex (TSC) [2,3]. The metastatic rate for ChRCC is much lower than for other RCC subtypes (approximately 5%) [4,5]. However, the lack of specific therapies targeting ChRCC tumor biology results in a significant unmet clinical challenge [6].

ChRCC is genetically distinct from other RCC subtypes such as clear cell renal cell carcinoma (ccRCC). Most ChRCCs harbor widespread whole-chromosome losses and have a low somatic mutational burden [7,8]. Mutations in mitochondrial DNA (mtDNA) are more frequent in ChRCC than in any other TCGA tumor type, suggesting that dysfunction in the mitochondrial electron transport chain contributes to tumor biology [8,9,10]. These alterations align with the characteristic ultrastructure of ChRCC, in which mitochondria are frequently dysmorphic and show aberrant, often tubulovesicular cristae [11,12].

The molecular differences in ccRCC and ChRCC are also mirrored in their metabolic profiles. While ccRCC is defined by profound glycolytic reprogramming and lipid accumulation [13,14], ChRCC is characterized by a strong oxidative, mitochondria-driven metabolic program, with elevated expression of the Krebs cycle and electron transport chain genes, enhanced pyruvate dehydrogenase activity to drive oxidative phosphorylation, and increased AMPK signaling [15]. However, it is not known whether these distinct molecular features could be leveraged for biomarker discovery.

The circulating proteome reflects a variety of physiologic processes and disease states, and several measurable proteins have been shown to predict clinical outcomes in patients with RCC [16,17,18,19]. An aptamer-based proteomics assay, SomaScan, can simultaneously measure up to 11,000 circulating proteins. This platform for biomarker discovery could capture tumor-driven metabolic changes that may not be apparent at the tissue level alone [20,21]. The multiplexed assay has previously been employed for the discovery of diagnostic biomarkers, pathway interrogation, and prediction of treatment responses [22,23,24]. We aimed to evaluate whether ChRCC was associated with a distinct plasma proteomic signature that can predict ChRCC and whether this signature could be linked to underlying tumor biology.

We performed plasma proteomic profiling using SomaScan on 197 ccRCC and 18 ChRCC samples. This revealed differentially enriched proteins and upregulated pathways in ChRCC related to mitochondrial protein degradation, fatty acid β-oxidation, as well as aerobic respiration and respiratory electron transport. A subsequently developed mitochondria-restricted Least Absolute Shrinkage and Selection Operator (LASSO) model reliably distinguished ChRCC from ccRCC, and enrichment of the top mitochondrial proteins was validated in two independent cohorts. This approach provides insight into mitochondrial adaptation in metastatic ChRCC and demonstrates the feasibility of a plasma-based mitochondrial signature for detecting metastatic ChRCC.

## 2. Materials and Methods

### 2.1. Patient Cohorts

**Figure 1:**
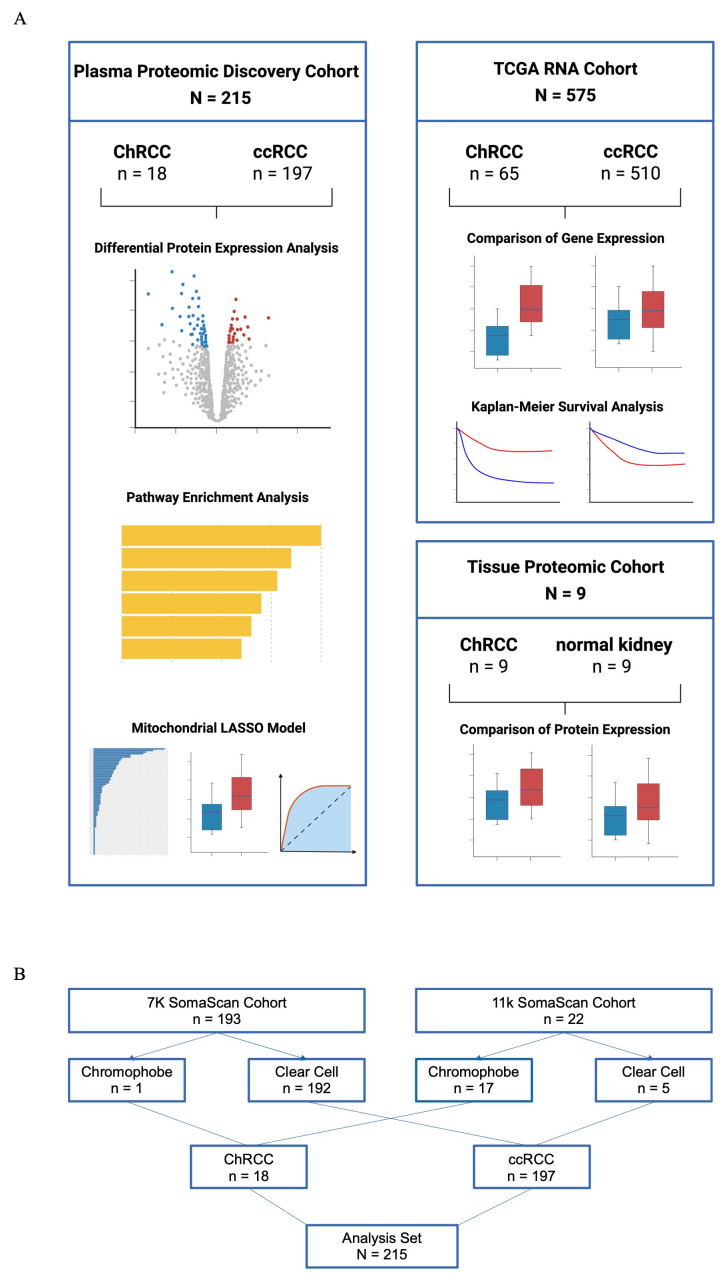
Overview of RCC patient cohorts: (**A**) Schematic view of conducted analyses per patient cohort. Based on findings from the plasma proteomic discovery cohort, proteins and corresponding genes of interest are further explored in a TCGA RNA cohort and a tissue-based proteomic cohort. (**B**) Cohort flow chart of ChRCC and ccRCC patients regarding the SomaScan assay used.

Three retrospective patient cohorts with ChRCC were analyzed (Figure 1A). Our primary proteomic discovery cohort consisted of a total of 215 patients, including 18 patients with ChRCC and 197 with ccRCC. All patients had pathologically confirmed diagnoses. As part of an ongoing prospective biospecimen banking initiative, plasma samples had been collected through the Dana-Farber/Harvard Cancer Center (DF/HCC) Kidney Cancer SPORE biorepository. Patients with ChRCC were included in this study if they had plasma samples available, regardless of the timepoint within their disease course. For ccRCC, patients with metastatic disease were preferentially selected for inclusion when samples were collected prior to the initiation of systemic therapy. Findings from the discovery cohort were further explored in the RNA cohort from TCGA comparing ChRCC and ccRCC, as well as the tissue-based proteomics cohort previously published by Xiao and colleagues comparing ChRCC and matched healthy kidney [25].

### 2.2. SomaScan Proteomics

Protein concentrations were quantified using the SomaScan platform (SomaLogic, Boulder, CO, USA). Normalization and calibration were performed according to SomaLogic’s internal protocols, yielding relative fluorescence units (RFU) for each analyte [20]. The 11k and 7k assays were used for data generation (Figure 1B). Data from the 11K SomaScan assay were harmonized to the 7K-protein space by leveraging a bridging framework for human plasma provided by SomaLogic within the SomaDataIO [26] package (version 6.4.0) using R Studio (R version 4.4.2). The 11K assay was designed to preserve the same precision, reproducibility, and sensitivity as the 7K panel, and independent evaluations using technical replicates have demonstrated high correlation and low variability between the two assay versions. Harmonization relied on analyte-specific scalars for linear transformation of RFUs into a comparable signal space, resulting in a 7K-equivalent dataset suitable for downstream analyses [27].

### 2.3. Differential Expression

Protein intensities were log_2_-transformed, and differential expression was performed using the limma package (version 3.62.2) in R (version 4.4.2). A design matrix contrasting ChRCC vs. ccRCC was used to fit linear models with empirical Bayes moderation. Proteins with absolute log_2_ fold change (|log_2_FC|) ≥ 1 and false discovery rate (FDR) ≤ 0.05 were defined as differentially expressed.

### 2.4. Pathway Enrichment

KEGG (Kyoto Encyclopedia of Genes and Genomes) and Reactome pathway enrichment analyses were performed using the clusterProfiler (version v4.14.6) and enrichR (version 3.4) packages. Significant pathways were defined by FDR-adjusted *p* ≤ 0.05. Top pathways were visualized using ggplot2 (version 4.0.0).

### 2.5. Mitochondrial LASSO Model

We implemented bootstrap-based LASSO feature selection with out-of-bag (OOB) validation across 100 iterations (glmnet, version 4.1-10; caret, version 7.0-1). In each iteration, data were resampled with replacement to generate a training set, and performance was assessed on OOB test samples. Proteins selected in at least 1/3 of the bootstraps were retained. Final diagnostic scores were computed as weighted sums of scaled expression values multiplied by model coefficients. AUROC values were calculated for each iteration, and feature selection frequencies were summarized. Model stability of AUC, sensitivity, and specificity was assessed across 100 bootstrap iterations. Multivariable logistic regression was performed to evaluate whether the mitochondrial LASSO score remained an independent predictor of ChRCC after adjusting for clinical covariates, including prior systemic therapy, IMDC risk category, and the presence of sarcomatoid/rhabdoid features. Sensitivity analyses further evaluated model performance in clinically defined subsets of metastatic cases and treatment-naïve patients.

### 2.6. Survival Analysis

Overall survival (OS) was evaluated using Kaplan–Meier curves and a two-sided log-rank test. OS was defined as the time from plasma sample collection to either death or loss of follow-up. Patients were stratified into low- and high-expression groups for proteins of interest selected by the LASSO model. For multi-protein signatures, optimal cut points were determined using maximally selected rank statistics based on the log-rank test to define risk groups, whereas single-protein measurements were dichotomized using the median. Univariable Cox proportional hazards models were used to estimate hazard ratios (HRs) and 95% confidence intervals (95% CI). Survival analyses and visualizations were performed using the survival (version 3.8-3) and survminer (version 0.5.1) R packages.

### 2.7. Survival Analysis Using TCGA Chromophobe Renal Cell Carcinoma

Overall survival analyses for the RNA cohort were performed using clinical and RNA-sequencing data from chromophobe renal cell carcinoma (TCGA-KICH) obtained from The Cancer Genome Atlas via the Genomic Data Commons utilizing the TCGAbiolinks (version 2.34.1) R package. Gene expression quantification was based on the STAR–Counts workflow, and transcript abundance values were extracted from the unstranded Transcripts Per Million (tpm_unstrand) assay. Only primary tumor samples (TCGA sample type code “01”) were included. Ensembl gene identifiers were mapped to HGNC gene symbols.

Clinical data were retrieved using GDCquery_clinic function from the TCGAbiolinks package (version 2.34.1). Overall survival time was defined as the number of days from diagnosis to death or to last follow-up for censored patients, and was then converted to months. For each gene, patients were stratified into low- and high-expression groups using a median split.

### 2.8. Tissue-Based Proteomics Cohort

Additionally, we analyzed tissue-based proteomic data from Xiao et al., which included ChRCC samples and matched adjacent normal kidney tissue [25]. Protein abundance between ChRCC and matched normal samples was compared using the Wilcoxon signed-rank test, a non-parametric test for paired data. Statistical significance was assessed using a two-sided test with a predefined alpha level of 0.05 (*p* < 0.05).

## 3. Results

### 3.1. Patient Cohorts

The patient characteristics for the proteomic discovery cohort, comprising 18 ChRCC, of whom 16 patients had metastatic disease, and 197 metastatic ccRCC cases, are summarized in Table 1. The majority of patients were male (77.7%), with a median age at blood collection of 61 years (interquartile range [IQR], 56–69 years). Most patients had metastatic disease (99.1%) at the time of sample collection. Eleven ChRCC patients (61.1%) had received prior therapies, compared with four patients with ccRCC (2.0%). At the time of blood collection, pre-treated ChRCC patients were on their first to fourth line of therapy and had previously received single agents or combinations of immune checkpoint inhibitors (ICIs), tyrosine kinase inhibitors (TKIs), mTOR inhibitors, and VEGF inhibitors. Pre-treated ccRCC patients had received one to five prior lines of therapy including single agents or combinations of ICIs, TKIs, and mTOR inhibitors. According to the IMDC risk classification, 18.6% had favorable, 48.4% intermediate, and 13.5% poor risk disease, with the remaining patients either not applicable or missing data. Sarcomatoid or rhabdoid features were present in 21.9% of cases. The characteristics of both external cohorts have been described previously [8,25]. For the TCGA cohort, 65 patients were included in the survival analysis after merging expression and clinical data and excluding samples with missing survival information, of whom 9 died and 56 were censored at the time of analysis.

### 3.2. Metabolic Proteins Are Upregulated in ChRCC Plasma vs. ccRCC Plasma

**Figure 2:**
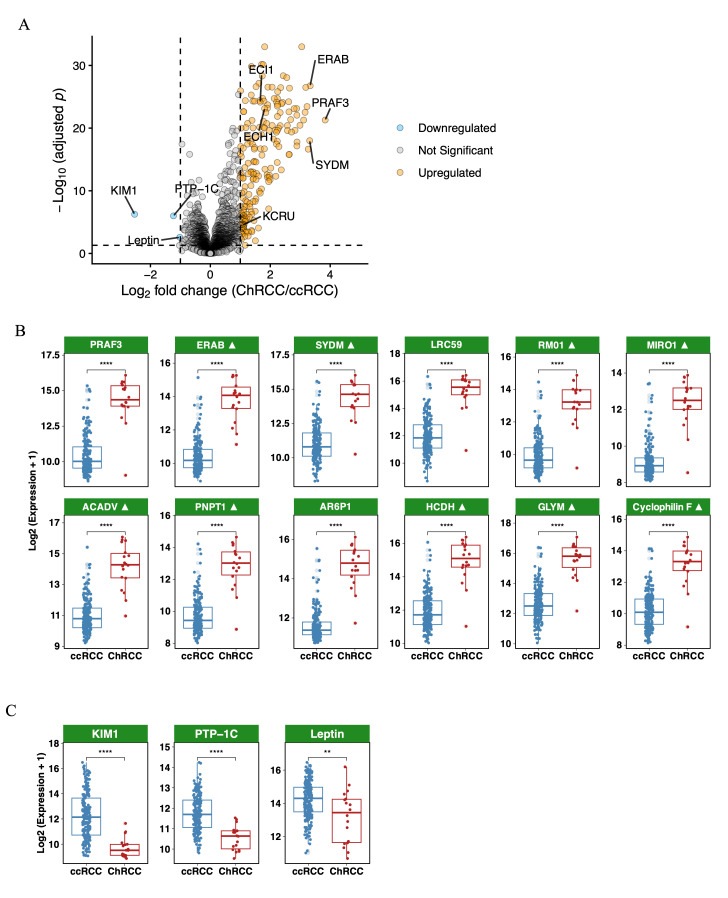
Proteomic landscape and pathway alterations in ChRCC compared with ccRCC: (**A**) Volcano plot showing differentially expressed proteins. A total of 206 proteins are significantly upregulated in ChRCC, while only three are downregulated, based on the criteria |log_2_FC| ≥ 1 and adjusted *p* value ≤ 0.05. (**B**) Boxplots illustrating the protein expression of the top 12 upregulated proteins in ChRCC vs. ccRCC. Symbol ▲ indicates mitochondrial proteins. (**C**) Boxplots illustrating the protein expression of three downregulated proteins in ChRCC vs. ccRCC. Statistical significance is indicated as: ** *p* < 0.01; **** *p* < 0.0001.

Among the 7289 quantified proteins, 209 were significantly altered between ChRCC and ccRCC (Figure 2A). Only three proteins were significantly downregulated in ChRCC relative to ccRCC: Kidney Injury Molecule-1 (KIM-1, gene name *HAVCR1*; log_2_FC = −2.5), Protein Tyrosine Phosphatase 1C (PTP-1C, gene name *PTPN6*; log_2_FC = −1.2), and leptin (LEP, gene name *LEP*; log_2_FC = −1.0), as shown in Figure 2C. The downregulation of KIM-1 is consistent with known subtype-specific expression profiles, as KIM-1 is highly expressed in ccRCC and serves as a well-established diagnostic marker for this subtype [19,28], whereas ChRCC does not typically overexpress KIM-1 [29].

**Figure 3:**
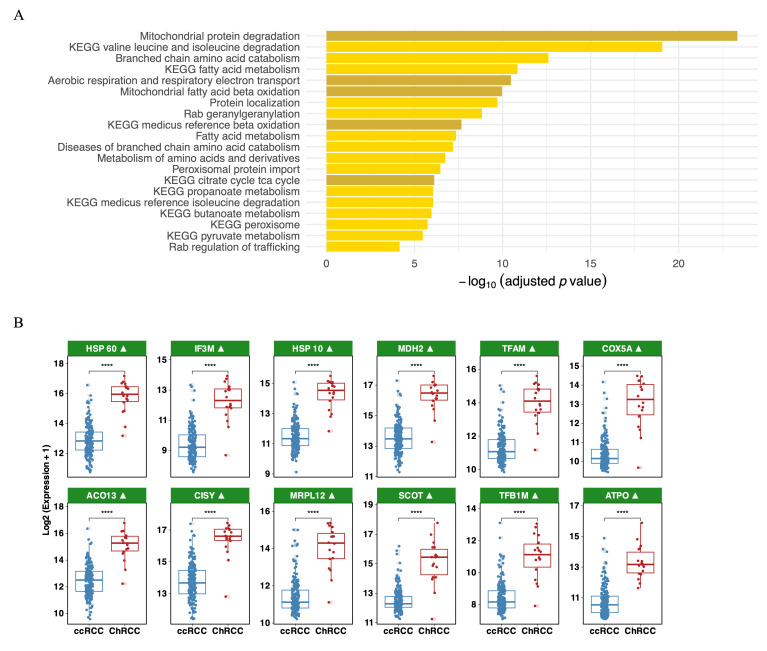
Associated pathways with ChRCC in comparison to ccRCC: (**A**) Pathway enrichment of upregulated proteins in ChRCC plasma, highlighting mitochondrial oxidative pathways (indicated in dark yellow). (**B**) Boxplots illustrating the expression of the additional top 12 upregulated mitochondrial proteins in ChRCC vs. ccRCC (non-overlapping with Figure 2B). Symbol ▲ indicates mitochondrial proteins. Statistical significance is shown as: **** *p* < 0.0001.

**Figure 4:**
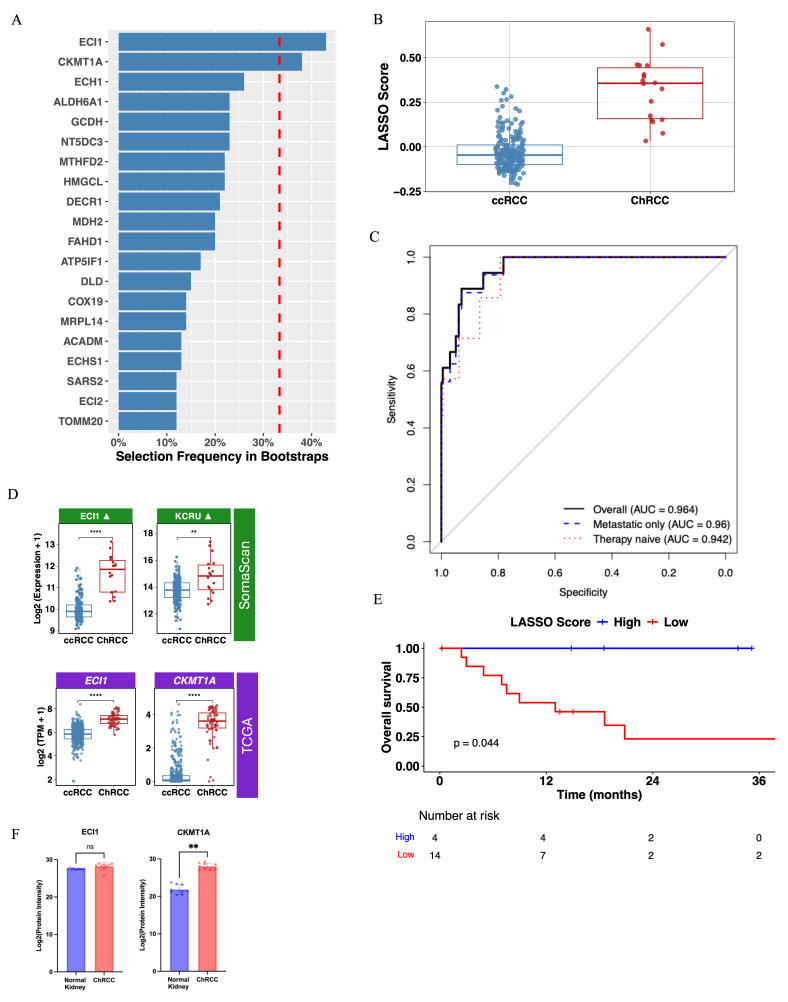
Identification, evaluation, and prognostic relevance of LASSO-selected mitochondrial proteins in ChRCC: (**A**) Selection frequency of the top 20 proteins identified by bootstrap-based LASSO. Bars represent the frequency with which each protein is selected across bootstrap iterations. The red dashed line marks the 33% threshold, indicating proteins selected in at least one-third of the bootstraps. (**B**) Boxplot showing the distribution of LASSO scores across different sample groups. LASSO scores are calculated using a linear combination of selected protein features weighted by their coefficients from the bootstrap-based LASSO model, as indicated by the formula: LASSO Score = ECI1 × 0.142 + CKMT1A × 0.018. Each point represents an individual sample, and the jittered points visualize the spread of scores within each group. The plot illustrates how the combined contribution of selected proteins differentiates the sample groups based on the LASSO model. (**C**) Receiver Operating Characteristic (ROC) curve showing the model’s classification performance in the overall cohort and subsets of patients with metastatic disease and treatment naïve patients. The Area Under the ROC Curve (AUC) quantifies the overall model performance. (**D**) Boxplots illustrating the protein expression of LASSO-selected proteins in ChRCC vs. ccRCC (green banner). Symbol ▲ indicates mitochondrial proteins. The lower panels show the corresponding mRNA expression levels from the TCGA dataset (purple banner). (**E**) Kaplan–Meier survival curves of patients stratified by the optimal cutoff of the Mitochondrial Score, identified using bootstrap-based LASSO. (**F**) Boxplot visualizing the comparison of tissue-based protein expression of ECI-1 and CKMT1A between ChRCC and matched normal kidney from the tissue proteomic cohort. Statistical significance is shown as: ns, not signficant; ** *p* < 0.01; **** *p* < 0.0001.

The proteins upregulated in ChRCC plasma were dominated by mitochondrial metabolic enzymes, consistent with the known accumulation of abnormal mitochondria in ChRCC cells (Figure 2B and Table 2; Figure 3B and Table 3). The upregulated proteins included essential β-oxidation enzymes such as enoyl-CoA hydratase 1 (ECH1), acyl-CoA dehydrogenase very long chain (ACADVL), and enoyl-CoA delta-isomerase 1 (ECI1), indicating intensified long-chain fatty acid degradation. Enzymes involved in ketone body turnover, including succinyl-CoA:3-ketoacid CoA transferase 1 (OXCT1) and acetyl-CoA acetyltransferase 1 (ACAT1), were also elevated. One-carbon metabolism and NADH-generating reactions were enriched, with increased expression of methylenetetrahydrofolate dehydrogenase 2 (MTHFD2), methylenetetrahydrofolate dehydrogenase 2-like (MTHFD2L), serine hydroxymethyltransferase 2 (SHMT2), fumarylacetoacetate hydrolase domain-containing 1 (FAHD1), and dihydrolipoamide dehydrogenase (DLD). Multiple components of the mitochondrial translation machinery (mitochondrial ribosomal protein L10 (MRPL10), MRPL12, MRPL14), the mitochondrial import complex (translocase of outer mitochondrial membrane 20 (TOMM20)), and respiratory chain assembly proteins (synthesis of cytochrome c oxidase 2 (SCO2), NADH:ubiquinone oxidoreductase subunit B6 (NDUFB6)) were significantly elevated. Mitochondrial stress-response proteins, including peroxiredoxin 3 (PRDX3), heat shock protein family D member 1 (HSPD1), heat shock protein family E member 1 (HSPE1), and glutathione S-transferase kappa 1 (GSTK1), were strongly upregulated. Creatine and energy-buffering pathways were also prominently represented, with creatine kinase, mitochondrial 1A (CKMT1A, shown in Figure 4D), and glycine amidinotransferase (GATM) increased in ChRCC plasma. CKMT1A protein level was also shown to be significantly higher in ChRCC tumors when compared with adjacent normal kidney tissue (Figure 4F).

Overall, the ChRCC plasma proteome reflects a mitochondrial-rich metabolic signature that is measurable in circulation.

### 3.3. Pathway Enrichment Analysis Reveals a Mitochondrial Signature in ChRCC Plasma

Pathway enrichment analyses revealed that mitochondrial and metabolic pathways dominate the ChRCC plasma proteome. Fatty acid β-oxidation, valine/leucine/isoleucine degradation, mitochondrial protein degradation, and oxidative phosphorylation were the most significantly enriched pathways (Figure 3A). Long-chain fatty acid metabolic pathways were strongly represented. Elevated branched-chain amino acid (BCAA) degradation was also observed. Enrichment of mitochondrial protein import, translation, and assembly pathways, including TOMM complex activity, ribosomal protein synthesis, and respiratory chain assembly, may reflect enhanced mitochondrial biogenesis. The breadth of these enriched pathways indicates that ChRCC is characterized not merely by mitochondrial markers, but by a broad repertoire of metabolic infrastructure proteins supporting oxidative, amino acid-based, and fatty acid-derived respiration.

### 3.4. Development of a Model for ChRCC

Based on the strong mitochondrial signature in ChRCC, we constructed a mitochondria-restricted LASSO model limited to MitoCarta-annotated proteins. Across 100 bootstrap iterations, enoyl-CoA delta isomerase 1 (ECI1) and creatine kinase, mitochondrial 1A (CKMT1A) were consistently selected as the most informative proteins (Figure 4A), surpassing the 33% selection frequency threshold. LASSO scores were calculated as a linear combination of the selected protein features weighted by their coefficients: LASSO Score = ECI1 × 0.142 + CKMT1A × 0.018, which clearly differentiated ChRCC from other sample groups (Figure 4B). The final model achieved an AUROC of 0.964, suggesting that this dyad of mitochondrial proteins could distinguish ChRCC from ccRCC (Figure 4C). We next evaluated the performance of the individual proteins relative to the composite LASSO score. While ECI1 demonstrated strong individual predictive power (AUC = 0.95), the composite model (AUC = 0.96) provided numerically better discrimination (Supplementary Figure S1A). Model stability was further evaluated across 100 bootstrap iterations. The median AUROC was 0.87, the median sensitivity was 1.00, and the median specificity was 0.91. However, the IQR for specificity was 0.96, with the full range of performance metrics spanning 0.00–1.00, reflecting the limited size of the ChRCC cohort (Supplementary Figure S1B, Supplementary Table S1A).

To estimate the model’s generalizability to unseen data, we performed 100 bootstrap iterations with out-of-bag (OOB) evaluation. In each iteration, the model was trained on a bootstrap sample and tested on the remaining held-out samples. This yielded a mean OOB AUROC of 0.757.

Multivariable logistic regression analyses were used to determine whether the protein score remained an independent predictor of ChRCC when accounting for key clinical variables. As shown in Supplemental Table S1B and the corresponding forest plot (Supplemental Figure S1C), the signature remained a highly significant independent predictor (*p* < 0.001) with a high odds ratio when adjusting for prior systemic therapy, IMDC risk category, and the presence of sarcomatoid or rhabdoid features. Furthermore, sensitivity analyses were performed to evaluate the LASSO score in different clinical subsets. When restricted to metastatic cases (n = 213) and to treatment-naïve patients (n = 200), the model achieved AUCs of 0.960 and 0.942, respectively. Overlayed ROC curves demonstrated consistently high discrimination across these subsets and the overall cohort (Figure 4C).

In both the proteomic discovery and TCGA cohorts, ECI-1 and CKMT1A expressions were significantly higher in ChRCC compared to ccRCC (Figure 4D).

### 3.5. Survival Associations of Mitochondrial Proteins in ChRCC

Survival analysis indicated the potential prognostic relevance of the LASSO-selected mitochondrial score. A high LASSO score was associated with worse overall survival in metastatic ChRCC in the proteomic discovery cohort (*p* = 0.044; Figure 4E). When each protein from the score was analyzed separately using a median split, no significant differences in overall survival were observed between high- and low-expression groups for either CKMT1A or ECI-1 in the proteomic discovery cohort or in TCGA (Supplemental Figure S2A–D).

Kaplan–Meier survival analyses on additional mitochondrial-related proteins selected by LASSO, which were upregulated in ChRCC compared to ccRCC using TCGA-KICH data, revealed several proteins with statistically significant associations with overall survival (*p* < 0.05). For instance, acyl-CoA synthetase family member 2 (ACSF2) (HR = 9.63, *p* = 0.0089), 5′-nucleotidase domain containing 3 (NT5DC3) (HR = 10.79, *p* = 0.0051), and dihydrolipoamide dehydrogenase (DLD) (HR = 9.95, *p* = 0.0079) demonstrated strong prognostic effects, with lower expression generally associated with better survival.

## 4. Discussion

This study, to our knowledge, provides initial evidence that the mitochondrial phenotype of ChRCC is reflected in a measurable plasma proteomic signature. Proteins central to fatty acid β-oxidation (ECH1, ACADVL, ECI1), ketone body metabolism (OXCT1, ACAT1), one-carbon and NADH-generating metabolism (MTHFD2, MTHFD2L, SHMT2, FAHD1, DLD), mitochondrial translation and import (MRPL10, MRPL12, MRPL14, TOMM20, SCO2, NDUFB6), mitochondrial stress response (PRDX3, HSPD1, HSPE1, GSTK1), and creatine/energy buffering (CKMT1A, GATM) were strongly elevated in the plasma from patients with ChRCC. These elevations suggest intensified fatty acid oxidation, amino acid-linked metabolism, and mitochondrial energy production in ChRCC. The strong and recurrent enrichment of β-oxidation enzymes, creatine kinase, and other mitochondrial proteins may reflect a high oxidative metabolic state, supporting energy efficiency and redox balance.

Our findings are consistent with prior studies demonstrating that mitochondrial metabolism is a defining feature of ChRCC. In vivo metabolic tracing in patients has shown that ChRCC tumors exhibit preserved oxidative metabolism and impaired complex I activity relative to adjacent healthy kidney tissue, distinguishing them from ccRCC and connecting mitochondrial function to chromophobe biology [8,30]. Complementary metabolomic and proteomic analyses of primary ChRCC tumors have highlighted alterations in mitochondrial pathways, redox balance, and lipid metabolism [25,31], supporting a central role for mitochondrial remodeling in this disease. In line with the high frequency of mtDNA mutations affecting components of the respiratory chain in ChRCC [8,9,10], the enrichment of fatty acid β-oxidation enzymes and ketone body metabolism observed in our analysis may reflect metabolic adaptation in response to altered mitochondrial bioenergetics.

Notably, most prior metabolic and proteomic studies have focused on primary ChRCC tumors, whereas the biology of metastatic ChRCC remains poorly defined. Our plasma proteomic data, mainly derived from patients with metastatic disease, identify systemic enrichment of mitochondrial-associated pathways, connecting prior tissue-based observations to the circulating proteome. Owing to the overall limited number of patients with metastatic ChRCC, all patients with available plasma in the DF/HCC biobank were included in this study, irrespective of the timing of plasma collection during their disease course. Since patient consent for sample collection was primarily obtained through medical oncology, the ChRCC subgroup predominantly comprised patients with metastatic rather than localized disease (16 vs. 2 patients). In contrast, a substantially larger patient pool was available for ccRCC, allowing selection of samples collected prior to initiation of systemic therapy, while for ChRCC, 61% had received prior systemic treatment. Nevertheless, the observed enrichment of redox and ferroptosis-related proteins in ChRCC is consistent with recent findings linking mitochondrial metabolism, oxidative stress handling, and ferroptosis sensitivity in renal cancers [30,31]. Furthermore, sensitivity analyses suggest that the mitochondrial protein signature is maintained across different disease stages and prior treatment histories and might therefore be a tumor-intrinsic feature independent of disease burden or prior treatment effects. Together, these data indicate that mitochondrial adaptation characteristics of ChRCC may not be confined to the primary tumor and may also be reflected systemically in metastatic disease.

The mitochondria-restricted LASSO model suggests that this oxidative metabolic phenotype is not only biologically informative but may also have potential diagnostic relevance. The discriminatory performance of ECI1 combined with CKMT1A supports mitochondrial metabolism itself as a promising biomarker axis warranting further clinical investigation. From a clinical perspective, such a plasma protein signature could help distinguish ChRCC from ccRCC. For example, patients classified as ccRCC with low KIM-1 could be evaluated for the ChRCC signature to reduce misdiagnosis. Both proteins are detectable using commercially available ELISAs, enabling straightforward evaluation in future studies. Such studies could also determine whether longitudinal changes in the signature reflect disease progression or response to therapy.

The integration of transcriptomic survival analysis with SomaScan proteomics highlights a coherent set of mitochondrial proteins that appear to contribute to poor outcomes in ChRCC. High TCGA–KICH expression of genes involved in multiple metabolic domains, such as fatty acid β-oxidation (DECR1, ECI2, HSD17B10), acyl-CoA and lipid metabolism (GCDH, ACSF2, ACOT13, MCEE), and mitochondrial nucleotide metabolism (NT5DC3, NT5M), was consistently associated with reduced overall survival. The corresponding proteins were upregulated in patient plasma. Several genes involved in mitochondrial energy production and protein homeostasis, such as DLD, MRPL14, HSPA9, and HSPD1, also show links to poor prognosis, suggesting that enhanced mitochondrial translation, chaperone activity, and mitochondrial dehydrogenase function may support tumor metabolic fitness for survival. Additional contributors, including RAB24 and RHOT1, point toward roles in mitochondrial autophagy and organelle trafficking that may further promote tumor survival. Collectively, these findings may indicate that ChRCC with poor clinical outcomes exhibit coordinated upregulation of mitochondrial pathways associated with metabolic flexibility, stress resistance, and bioenergetic output, raising the possibility that mitochondrial metabolism could contribute to aggressive disease behavior and represent a potential therapeutic vulnerability.

These findings encourage further translational work, including validation in larger, independent cohorts to confirm their clinical utility, targeted quantification using mass spectrometry or ELISA, and integration with tumor-level transcriptomic and metabolomic data. Ultimately, mitochondrial plasma signatures may provide minimally invasive tools for detecting, subtyping, and monitoring therapeutic responses in ChRCC within the context of a clinical trial.

## 5. Conclusions

In summary, high-throughput plasma proteomics reveals that metastatic ChRCC is characterized by a distinctive mitochondrial metabolic signature, marked by enhanced β-oxidation, amino acid oxidation, mitochondrial biogenesis, and redox buffering. A compact mitochondrial classifier derived from this signature offers a mechanistically grounded plasma-based tool for distinguishing ChRCC from other RCC subtypes. These findings enhance our understanding of ChRCC metabolism and lay the groundwork for mitochondrial biomarker development in renal oncology.

## Figures and Tables

**Table 1:** Baseline demographics and clinicopathological characteristics of the proteomic discovery cohort. Patient characteristics are outlined for the overall cohort and stratified by histologic subtype. IMDC, International Metastatic RCC Database Consortium.

	Cohorts		Total
	Chromophobe RCC	Clear-Cell RCC	
	n (%)	n (%)	N (%)
Total	18 (100)	197 (100)	215 (100)
Sex			
Female	3 (16.7)	45 (22.8)	48 (22.3)
Male	15 (83.3)	152 (77.2)	167 (77.7)
Disease Setting			
Localized Disease	2 (11.1)	0 (0)	2 (0.9)
Metastatic Disease	16 (88.9)	197 (100)	213 (99.1)
Previous Therapies			
Yes	11 (61.1)	4 (2.0)	15 (7.0)
No	7 (38.9)	193 (98.0)	200 (93.0)
IMDC Risk Group			
Favorable	3 (16.7)	37 (18.8)	40 (18.6)
Intermediate	7 (38.9)	97 (49.2)	104 (48.4)
Poor	4 (22.2)	25 (12.7)	29 (13.5)
Not Applicable	3 (16.7)	0 (0)	3 (1.4)
Missing	1 (5.6)	38 (19.3)	39 (18.4)
Sarcomatoid/Rhabdoid Features			
Present	6 (33.3)	41 (20.8)	47 (21.9)
Not Present	12 (66.7)	156 (79.2)	168 (78.1)
	**Median (IQR)**	**Median (IQR)**	**Median (IQR)**
Age at Blood Collection (years)	59.5 (53.8–66)	62 (56–69)	61 (56–69)

**Table 2:** Top 12 upregulated proteins in ChRCC vs. ccRCC.

Protein	Gene Symbol	Full Gene Name	log_2_FC	Function
PRAF3	*ARL6IP5*	ADP Ribosylation Factor Like GTPase 6 Interacting Protein 5	3.83	ER stress-related protein; involved in apoptosis and vesicle trafficking
ERAB	*HSD17B10*	Hydroxysteroid 17-Beta Dehydrogenase 10	3.33	Mitochondrial enzyme in fatty-acid and isoleucine metabolism; linked to neurodegeneration
SYDM	*DARS2*	Aspartyl-tRNA Synthetase 2, Mitochondrial	3.32	Mitochondrial translation; essential for mtDNA-encoded protein synthesis
LRC59	*LRRC59*	Leucine-Rich Repeat Containing Protein 59	3.27	ER/ER-mitochondria contact site protein; facilitates protein trafficking and mRNA import
RM01	*MRPL1*	Mitochondrial Ribosomal Protein L1	3.23	Component of the mitochondrial ribosome large subunit; mitochondrial translation
MIRO1	*RHOT1*	Ras Homolog Family Member T1	3.19	Mitochondrial trafficking GTPase; regulates mitochondrial dynamics and movement
ACADV	*ACADVL*	Acyl-CoA Dehydrogenase Very Long Chain	3.16	First step of mitochondrial β-oxidation of very-long-chain fatty acids
PNPT1	*PNPT1*	Polyribonucleotide Nucleotidyltransferase 1	3.11	Mitochondrial RNA processing and import; essential for mtRNA stability
AR6P1	*ARL6IP1*	ADP Ribosylation Factor Like GTPase 6 Interacting Protein 1	3.05	ER membrane protein regulating vesicle transport and apoptosis
HCDH	*HADH*	Hydroxyacyl-CoA Dehydrogenase	2.99	Mitochondrial β-oxidation of short/medium-chain fatty acids
GLYM	*SHMT2*	Serine Hydroxymethyltransferase 2	2.89	Mitochondrial one-carbon metabolism; critical for nucleotide synthesis
Cyclophilin F	*PPIF*	Peptidylprolyl Isomerase F	2.89	Regulates mitochondrial permeability transition pore; apoptosis control

**Table 3:** Twelve upregulated mitochondrial proteins in ChRCC vs. ccRCC (non-overlapping with Table 2).

Protein Name	Gene Symbol	Full Gene Name	log2FC	Function
HSP 60	*HSPD1*	Heat Shock Protein Family D (Hsp60) Member 1	2.89	Mitochondrial chaperonin essential for protein folding and complex assembly
IF3M	*MTIF3*	Mitochondrial Translation Initiation Factor 3	2.83	Regulates initiation of mitochondrial protein translation
HSP 10	*HSPE1*	Heat Shock Protein Family E (Hsp10) Member 1	2.79	Co-chaperonin with HSP60; assists folding of imported mitochondrial proteins
Malate dehydrogenase 2	*MDH2*	Malate Dehydrogenase 2	2.71	Enzyme in the TCA cycle converting malate ↔ oxaloacetate
TFAM	*TFAM*	Mitochondrial Transcription Factor A	2.68	Essential for mtDNA packaging, replication, and transcription
COX5A	*COX5A*	Cytochrome c Oxidase Subunit 5A	2.64	Subunit of Complex IV (cytochrome c oxidase); regulates OXPHOS activity
ACO13	*ACOT13*	Acyl-CoA Thioesterase 13	2.63	Hydrolyzes fatty acyl-CoAs; regulates mitochondrial lipid metabolism
CISY	*CS*	Citrate Synthase	2.63	First and rate-limiting enzyme of the TCA cycle (oxaloacetate + acetyl-CoA → citrate)
39S ribosomal protein L12	*MRPL12*	Mitochondrial Ribosomal Protein L12	2.62	Component of mt-large ribosomal subunit; regulates mtRNA translation
SCOT	*OXCT1*	3-Oxoacid CoA-Transferase 1	2.61	Catalyzes ketone body utilization; key enzyme in ketolysis
TFB1M	*TFB1M*	Mitochondrial Transcription Factor B1	2.57	Required for mitochondrial rRNA methylation and ribosome assembly
ATPO	*ATP5PO*	ATP Synthase Peripheral Stalk Subunit O	2.57	Structural subunit of ATP synthase (Complex V); stabilizes F1–Fo coupling

↔: indicates a reversible reaction; →: indicates an irreversible/rate-limiting reaction.

## Data Availability

The data that support the findings of this study are available from the corresponding authors upon reasonable request.

## Supplementary Materials

The following supporting information can be downloaded at: https://www.mdpi.com/article/10.3390/cancers18061032/s1. Supplemental Figure S1: Validation and comparative performance of the mitochondrial LASSO model. (A) ROC curves showing the composite LASSO score and the individual protein predictors (ECI1 and CKMT1A). (B) Distribution of performance metrics of the two-analyte LASSO model across 100 bootstrap iterations, showing median, interquartile range, and full range for AUC, sensitivity, and specificity. (C) Forest plot depicting odds ratios and 95% confidence intervals for the LASSO score in multivariable logistic regression models adjusting for prior therapy, IMDC risk category, and sarcomatoid/rhabdoid features. ** *p* < 0.01; *** *p* < 0.001. Supplemental Figure S2: Kaplan–Meier survival curves stratified by LASSO-selected mitochondrial proteins in ChRCC patients from the discovery cohort and TCGA-KICH. (A) Kaplan–Meier survival curves of discovery cohort ChRCC patients stratified by CKMT1A protein. (B) Kaplan–Meier survival curves of discovery cohort ChRCC patients stratified by ECI-1 protein. (C) Kaplan–Meier survival curves of patients stratified by *CKMT1A* gene in TCGA-KICH. (D) Kaplan–Meier survival curves of patients stratified by *ECI-1* gene in TCGA-KICH. Supplemental Table S1: LASSO-score model performance and multivariable regression metrics. (A) Distribution of AUC, sensitivity, and specificity across 100 bootstrap iterations of the two-analyte LASSO model. (B) Multivariable logistic regression analysis for the prediction of ChRCC with the LASSO protein score adjusting for prior treatment, IMDC risk category, and sarcomatoid/rhabdoid features. AUC = area under the curve, IQR = interquartile range, OR = odds ratio, CI = confidence interval, and IMDC = international metastatic RCC database consortium.
