# Supplementary material for: A Mitochondrial Plasma Proteomic Signature Identifies Metastatic Chromophobe Renal Cell Carcinoma
Source: Cancers (Basel). 2026 Mar 23;18(6):1032. doi: 10.3390/cancers18061032 (PMC13025462; doi:10.3390/cancers18061032)
Supplement: Supplementary file 1 [file cancers-18-01032-s001.zip › cancers-4170703-supplementary.pdf]

A mitochondrial plasma proteomic signature identifies metastatic chromophobe renal cell carcinoma

Supplements

Authors: Clara Steiner[1,2], Tiegang Han[3], Steven Safi[3,7], Wafaa Bzeih[3,7], Hadi Mansour[3,4], Eddy Saad[1,5], Jessica F. Williams[3,6], Michelle S. Hirsch[3,7], Vinay K. Giri[5], Liliana Ascione[1,8,9], Yehonatan Elon[10], Adam P. Dicker[11], Yan Tang[3,7], Toni K. Choueiri[1,7], Elizabeth P. Henske[1,3,7‡], Wenxin Xu[1,7‡].

‡ Co-corresponding authors

1 Dana-Farber Cancer Institute, Boston, MA, USA

2 Department of Urology, University Hospital Leipzig, Germany

3 Brigham and Women's Hospital, Boston, MA, USA

4 Henry Ford Hospital, Detroit, MI, USA

5 Beth Israel Deaconess Medical Center, Boston, MA, USA

6 University of Utah School of Medicine, Salt Lake City, UT, USA

7 Harvard Medical School, Boston, MA, USA

8 Division of Early Drug Development, IEO, European Institute of Oncology, IRCCS, Milan, Italy

9 Department of Oncology and Hematology (DIPO), University of Milan, Milan, Italy

10 Oncohost, Binyamina-Giv'at Ada, Israel

11 Thomas Jefferson University, Philadelphia, PA, USA

A

B

C

Adjusted Odds Ratio for ChRCC Prediction

Supplemental Figure S1

A

B

C

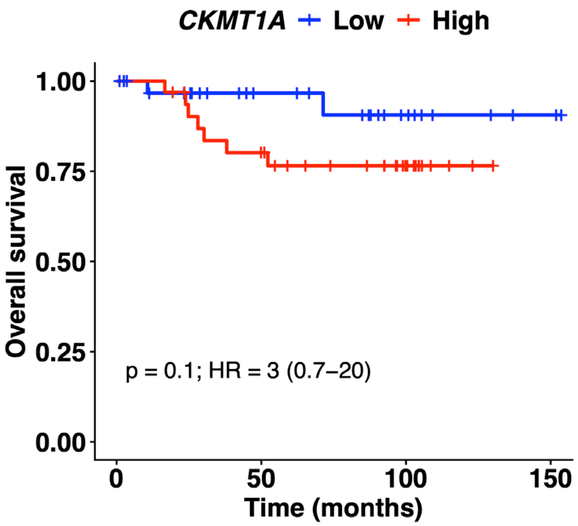

D

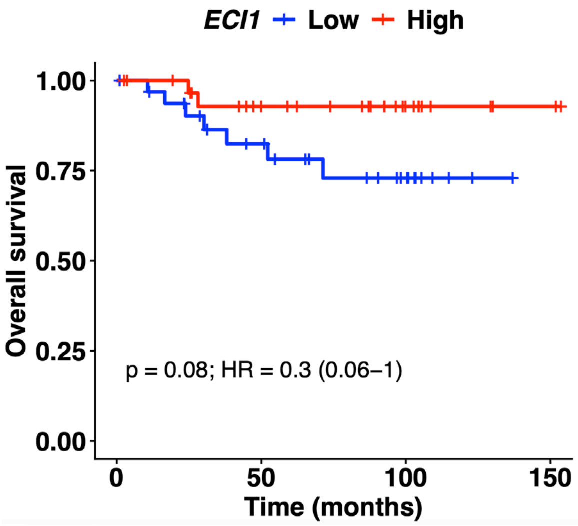

Supplemental Figure S2

A

**Metric**	**Median**	**IQR**	**Range**
AUC	0.87	0.48	[0.50, 1.00]
Sensitivity	1.00	0.00	[0.75, 1.00]
Specificity	0.91	0.96	[0.00, 1.00]

B

**Variable**	**OR**	**Lower CI**	**Upper CI**	**P-Value**
(Intercept)	0.01	0	0.05	< 0.001
LASSO Protein Score	1965702.41	5949.76	8508751099	< 0.001
Pre-treated (vs Naive)	21.6	3.51	179.06	0.0016
IMDC: Intermediate	0.46	0.04	5.05	0.50
IMDC: Poor	0.27	0	9.9	0.50
IMDC: Missing/NA	1.86	0.17	25.78	0.62
Sarcomatoid/Rhabdoid Features	4.68	0.56	44.83	0.15

Supplemental Table S1
